# Neurotoxicity at the Tides: A Call to Action on Marine Microplastics and Brain Health

**DOI:** 10.1111/ene.70181

**Published:** 2025-05-23

**Authors:** Raffaele Marfella, Ulf Kallweit

**Affiliations:** ^1^ Department of Advanced Medical and Surgical Sciences University of Campania “Luigi Vanvitelli” Caserta Italy; ^2^ Pollution and Cardiovascular Diseases Research Center University of Campania “Luigi Vanvitelli” Caserta Italy; ^3^ Faculty of Health University Witten/Herdecke Witten Germany; ^4^ Center for Biomedical Education and Research University Witten/Herdecke Witten Germany

**Keywords:** brain health, cognitive disorders, environmental influences, marine microplastics, neurological disabilities, neurotoxicity

The ubiquity of plastic waste in our oceans has long served as a stark reminder of human impact on the environment and the unreflective approach to nature. But a more insidious threat is now surfacing—literally. Microplastics (MPs), once considered primarily a marine ecological issue, are now increasingly linked to human health concerns, more precisely to brain health. Recent findings by Makwana et al. published in this issue of the *European Journal of Neurology*, mark a critical turning point: the demonstration of a population‐level association between exposure to marine microplastics and neurological and functional disabilities [1]. In their analysis of 218 coastal counties in the United States, the authors observed a striking pattern. Communities exposed to very high levels of marine microplastics showed a significantly elevated prevalence of cognitive, mobility, self‐care, and independent living disabilities—even after adjusting for age, socioeconomic vulnerability, comorbidities, and access to healthcare. They also accounted for other exposures, such as air pollution. While causation cannot be established from this cross‐sectional data, the consistency of associations and biological plausibility make the findings difficult to ignore [1]. Microplastics—defined as plastic particles smaller than 5 mm—are not benign environmental remnants. Experimental studies have shown that they can cross the intestinal barrier, enter the bloodstream, and penetrate the blood–brain barrier (BBB) [2, 3]. Once in neural tissue, microplastics can trigger oxidative stress, disrupt neurotransmitter systems, and activate pro‐inflammatory cascades [4]. Most concerning, they appear to promote the aggregation of misfolded proteins such as α‐synuclein and amyloid‐β, which are hallmark pathologies in Parkinson's and Alzheimer's diseases, respectively [5, 6]. The implications for neurology are profound. For decades, environmental risk factors such as delicate particulate matter (PM2.5) have been linked to stroke, cognitive decline, and neurodevelopmental disorders. The mechanistic parallels between air pollution and microplastic exposure—both of which involve systemic inflammation, blood–brain barrier (BBB) disruption, and proteinopathy—suggest that we may be witnessing the emergence of a new environmental neurotoxin [4, 7]. Recent evidence underscores the urgency of this issue. In a 2024 *New England Journal of Medicine* study, micro‐ and nanoplastics were identified in atherosclerotic plaques and were associated with increased cardiovascular events, including stroke—further linking plastic exposure to neurovascular injury [8]. Even more compelling, a 2025 *study in Nature Medicine* by Nihart and Campen et al. demonstrated microplastic accumulation in human post‐mortem brain tissue, with significantly higher concentrations in individuals diagnosed with dementia [9]. These human findings corroborate animal studies and raise the possibility that MPs may be contributing to neurodegenerative disease at the population level. See also Table S1. Yet, unlike PM2.5, microplastics are not monitored in clinical settings, and their exposure pathways are often invisible. Coastal populations may be disproportionately affected due to their greater reliance on seafood and potential groundwater contamination resulting from seawater intrusion [1, 10]. However, microplastics are now being detected in human blood, placenta, lungs, and brain tissue [3, 9]. This is not a coastal problem—it is a global one. There are, of course, significant limitations to the current study. The ecological design precludes the measurement of individual‐level exposure. Migration, dietary variation, and unmeasured confounders cannot be entirely ruled out. Also, cognitive and motor disabilities were only self‐reported. Still, the magnitude and consistency of the associations warrant attention. Neurological disability, especially in aging populations, represents a growing public health burden. Identifying modifiable environmental contributors is both urgent and essential. As neurologists, we are trained to detect patterns, interrogate causes, and advocate for prevention. Just as we now recognize vascular risk factors and air pollution as contributors to neurodegeneration, we must begin to reckon with the neurological impact of plastic pollution. This will require interdisciplinary collaboration among neuroscientists, environmental health experts, epidemiologists, and policymakers. We also need to fundamentally expand our concept of brain health and include environmental factors much more [11]. Makwana et al.'s work is a call to action. It reminds us that the brain does not exist in isolation from the world around us. Our environment shapes it—sometimes at the molecular level, sometimes on a societal scale. Marine microplastics, once a symbol of environmental neglect, are now emerging as potential agents of neurological harm. It is time to take the threat seriously.

## Author Contributions

**Raffaele Marfella:** conceptualization, writing – review and editing, writing – original draft. **Ulf Kallweit:** conceptualization, writing – original draft, writing – review and editing.

## Conflicts of Interest

The authors declare no conflicts of interest.

## Supporting information


Table S1.

## Data Availability

Data sharing is not applicable to this article as no new data were created or analyzed in this study.
