# Supplementary material for: Neurotoxicity at the Tides: A Call to Action on Marine Microplastics and Brain Health
Source: Eur J Neurol. 2025 May 23;32(5):e70181. doi: 10.1111/ene.70181 (PMC12100631; doi:10.1111/ene.70181)
Supplement: Supplementary file 1 — Table S1. [file ENE-32-e70181-s001.docx]

*Supplementary* Table 1. Pathophysiological Implications of Marine Microplastic Exposure on the Nervous System

| **Mechanism** | **Pathophysiological Effect** | **Neurological Consequence** | **Supporting Evidence** |
| --- | --- | --- | --- |
| **Ingestion and absorption** | Microplastics cross intestinal epithelium via paracellular transport and transcytosis | Systemic dissemination via bloodstream | (2,3) |
| **Blood-brain barrier (BBB) crossing** | Downregulation of tight junction proteins (occludin, claudin-5, ZO-1) allows MPs to cross into brain parenchyma | Increased CNS exposure to environmental toxins | (4,5) |
| **Neuroinflammation** | Microglial activation and cytokine release (TNF-α, IL-1β, IL-6, MCP-1) | Chronic neuroinflammation, glial scarring | (4,6) |
| **Oxidative stress** | Increased ROS, mitochondrial dysfunction, impaired antioxidant defenses | Neuronal damage and apoptosis | (4,7) |
| **Protein aggregation** | MPs promote misfolding and aggregation of α-synuclein and amyloid-β | Potential contribution to Parkinson’s and Alzheimer’s | (5,8,9) |
| **Neurotransmitter disruption** | Alteration of dopamine, serotonin, glutamate, GABA, and acetylcholine metabolism | Impaired cognition, mood, memory | (6,7) |
| **Peripheral inflammation** | Gut dysbiosis and endotoxemia (LPS elevation) reinforce central inflammatory response | Amplified BBB permeability and CNS injury | (4,10) |
| **Cognitive and functional decline** | Combined effects on neurons, glia, and vasculature | Dementia-like syndromes, motor impairment | (1,9) |

Legend: MPs = microplastics; BBB = blood-brain barrier; ROS = reactive oxygen species; CNS = central nervous system.
